# Supplementary material for: A high-resolution mRNA expression time course of embryonic development in zebrafish
Source: eLife. 2017 Nov 16;6:e30860. doi: 10.7554/eLife.30860 (PMC5690287; doi:10.7554/eLife.30860)
Supplement: Supplementary file 6. [file elife-30860-supp6.zip › biolayout-clusters-files/Cluster194-genes.html]

Cluster194


# Cluster194: Genes

| Ensembl ID | Gene Name | Chr | Start | End | Biotype |
| --- | --- | --- | --- | --- | --- |
| ENSDARG00000017844 | copz1 | 23 | 36241717 | 36255303 | protein\_coding |
| ENSDARG00000101061 | eif2a | 18 | 3455556 | 3474498 | protein\_coding |
| ENSDARG00000069977 | myg1 | 11 | 6899 | 12347 | protein\_coding |
| ENSDARG00000103464 | pggt1b | 8 | 411977 | 419003 | protein\_coding |
| ENSDARG00000006300 | psmd9 | 7 | 71757231 | 71765097 | protein\_coding |
| ENSDARG00000037188 | rpa2 | 16 | 4917299 | 4940678 | protein\_coding |
| ENSDARG00000017891 | tcp1 | 23 | 2482133 | 2504500 | protein\_coding |
